# 

**Published:** 1825-07

**Authors:** 


### THE LONDON MEDICAL AND PHYSICAL JOURNAL.

CONTAINING

ORIGINAL CORRESPONDENCE OF EMINENT PRACTITIONERS,

AND

CRITICAL ANALYSIS OF NEW WORKS

RELATING TO MEDICINE, SURGERY, MIDWIFERY, CHEMISTRY, PHARMACY, BOTANY, AND NATURAL HISTORY.

---

EDITED BY

RODERICK MACLEOD, M.D.

LICENTIATE OF THE COLLEGE OF PHYSICIANS OF LONDON; MEMBER OF THE MEDICO-CHIRURGICAL SOCIETY OF LONDON, AND OF THE ROYAL MEDICAL SOCIETY OF EDINBURGH; PHYSICIAN TO THE WESTMINSTER GENERAL DISPENSARY; AND TO THE SCOTTISH HOSPITAL; LATELY PHYSICIAN TO THE ROYAL INFIRMARY FOR CHILDREN; LECTURER ON THE PRACTICE OF PHYSIC AND MATERIA MEDICA, AT THE MEDICAL THEATRE, WINDMILL-STREET:

AND

JOHN BACOT, Esq.

MEMBER OF THE ROYAL COLLEGE OF SURGEONS; SURGEON TO THE ST. GEORGE'S AND ST. JAMES'S DISPENSARY; AND LATELY SURGEON TO HIS MAJESTY'S GRENADIER REGIMENT OF FOOT GUARDS.

---

VOL. LIV.

FROM JULY TO DECEMBER, 1825.

Et quoniam variant morbi, variabimus artes;
Mille mali species, mille salutis erunt.

45587

*LONDON:*

PRINTED FOR THE PROPRIETORS,

*By J. and C. Adlard, Bartholomew Close;*

PUBLISHED BY J. SOUTER, 73, ST. PAUL'S CHURCH-YARD; AND MAY BE HAD OF ALL BOOKSELLERS.

W1
LO 31

### MONTHLY CATALOGUE OF MEDICAL BOOKS.

*** *It having been hinted to us that gentlemen, sending copies of their Works, prefer having their titles given at length, it is our intention, in future, to comply with this suggestion: but it is to be observed, that no books can be entered on this List except those sent to us for the purpose;—finding, in the lists hitherto transmitted, that the names of works have frequently been given as published, which have not appeared for weeks, or even months, after.*

The Study of Medicine. Second Edition. By JOHN MASON GOOD, M.D. F.R.S. F.R.S.L. Member of the American Phil. Soc. and F. L. S. of Philadelphia. In five volumes.—London, 1825.

Small-Pox and Cow-Pox: comprehending a concise History of those Diseases, and a Comparison between Inoculation for Small-Pox and Vaccination, founded upon a Statistical Account of their Effects in Cambridge; with a Plan for the universal Extension of Vaccination, addressed to the Public. By J. JENNING CRIBB, Member of the Royal College of Surgeons.—Cambridge, 1825.

A Treatise on the most celebrated Mineral Waters of Ireland, &c. and of the Spa lately discovered at Brownstown, near Kilkenny; with plain Directions during the Use of the Mineral Waters, and an Account of those Diseases in which they are most useful. By MICHAEL RYAN, M.D. Member of the Royal College of Surgeons, London, &c.—Kilkenny, 1824.

A Letter to the Right Hon. W. Huskisson, M.P. President of the Board of Trade, on the Quarantine Bill. By A. B. GRANVILLE, M.D. F.R.S. &c. &c.—London, 1825.

A Letter to Sir Henry Halford, Bart. President of the Royal College of Physicians, proposing a Method of Inoculating the Small-Pox, which deprives it of all its Danger, and preserves all its Power of preventing the second Attack. By R. FERGUSON, M.D. Member of the Colleges of Physicians of London and Edinburgh.—London, 1825.

A Treatise on the different Methods of investigating the Diseases of the Chest, particularly Percussion and the use of the Stethoscope. Translated from the French of M. COLLIN, with an additional Preface, by W. N. RYLAND, M.D.—London, 1825.

☞ We strongly recommend this little volume to those desirous of becoming acquainted with the various methods of investigating the diseases of the chest lately adopted in France. It gives at once a concise and distinct account of the subject.

---

### NOTICE TO CORRESPONDENTS.

*The Papers of* Dr. VENABLES, Mr. ASHFORD, Mr. GRAY, Mr. EGAN, *and* Mr. MACKENZIE, *have been received.*

Mr. MONEY's, *and the continuation of* Dr. H. DAVIES's *Papers, in our next.*

Mr. JONES *will have the goodness to send his Paper as early in the month as possible.*

*We shall take an opportunity of answering by letter the proposal made by our Correspondent in Glasgow.*

Dr. KINGLAKE's *Papers and letter have been received: the former are unfit for publication, the latter requires no answer. We are sorry to find that our hint was not taken, and that we are thus compelled to be explicit.*

---

*Our readers will perceive that we have not given the usual* Historical Retrospect *on the present occasion. The extent to which it interfered with the other departments of the Journal, and the necessity which resulted of postponing numerous original Papers, have induced us to discontinue publishing a Proœmium for July, although we shall probably continue to do so at the beginning of each year.—We cannot let this opportunity pass of thanking most sincerely our numerous and increasing Correspondents, for their valuable contributions, which have enabled us to add greatly to the importance of the portion of our Journal devoted to Original Communications.*

